# New Variants of Porcine Epidemic Diarrhea Virus, China, 2011

**DOI:** 10.3201/eid1808.120002

**Published:** 2012-08

**Authors:** Wentao Li, Heng Li, Yunbo Liu, Yongfei Pan, Feng Deng, Yanhua Song, Xibiao Tang, Qigai He

**Affiliations:** Huazhong Agricultural University, Wuhan, People’s Republic of China (W. Li, H. Li; F. Deng, X. Tang, Q. He);; and Guangdong Wen’s Foodstuffs Group Company, Ltd., Xinxing, People’s Republic of China (Y. Pan, Y. Song)

**Keywords:** porcine epidemic diarrhea virus, prevalence, China, phylogeny, variant strain, viruses

## Abstract

In 2011, porcine epidemic diarrhea virus (PEDV) infection rates rose substantially in vaccinated swine herds. To determine the distribution profile of PEDV outbreak strains, we sequenced the full-length spike gene from samples from 9 farms where animals exhibited severe diarrhea and mortality rates were high. Three new PEDV variants were identified.

A member of the family *Coronaviridae,* genus *alphacoronavirus*, porcine epidemic diarrhea virus (PEDV) is an enveloped, single-stranded positive-sense RNA virus (*1*). PEDV is the major causative agent of porcine epidemic diarrhea, which is characterized by severe enteritis, vomiting, watery diarrhea, and weight loss. PEDV infections have a substantial detrimental effect on the swine industry because the mortality rates are high, especially in sucking piglets (*1*). The major structural gene of the 28-kb PEDV genome encodes the multifunctional virulence factor, spike (S), which is responsible for viral receptor binding, induction of neutralizing antibodies, and host cell fusion. The S gene sequences are a distinguishing feature of PEDV strains, which affect virulence and evolution (*2**–**4*).

The first confirmed PED case in the People’s Republic of China was reported in 1973. Almost 2 decades later, an oil emulsion, inactivated vaccine was developed and has since been in wide use throughout the swine industry in China. Until 2010, the prevalence of PEDV infection was relatively low with only sporadic outbreaks; however, starting in late 2010, a remarkable increase in PED outbreaks occurred in the pig-producing provinces. The affected pigs exhibited watery diarrhea (Figure 1, panels A, B), dehydration with milk curd vomitus (Figure 1, panel C), and thin-walled intestines (Figure 1, panel D) with severe villus atrophy and congestion (Figure 1, panels E, F). The disease progressed to death within a few days. Pigs of all ages were affected and exhibited diarrhea and loss of appetite with different degrees of severity, which were determined to be age dependent; 100% of suckling piglets became ill. Pigs >2 weeks of age experienced mild diarrhea and anorexia, which completely resolved within a few days (*5*). Morbidity and mortality rates were lower for vaccinated herds than for nonvaccinated herds, which suggests the emergence of a new PEDV field strain(s) for which the current vaccine, based on the CV777 strain, was partially protective. To identify the PEDV strain(s) responsible for the recent outbreak in China, we sequenced the full-length S gene of isolates obtained from diarrhea samples collected from pigs at 9 affected pig farms.

**Figure 1:**
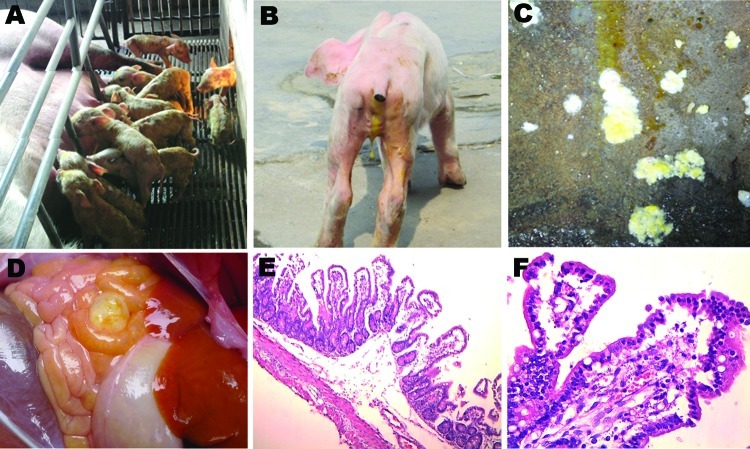
Clinical features of pigs infected with porcine epidemic diarrhea virus from pig farms in the People’s Republic of China, 2011. A) Litter of pigs infected with this virus, showing watery diarrhea and emaciated bodies. B) A representative emaciated piglet with yellow, water-like feces. C) Yellow and white vomitus from a representative sucking piglet. D) Thin-walled intestinal structure with light yellow water-like content. E) Congestion in the small intestinal wall and intestinal villi; desquamated epithelial cells from the intestinal villus (original magnification ×100). F) Congestion in the lamina propria of intestinal mucosa, and degeneration, necrosis, and desquamation of epithelial cells of the intestinal villi (original magnification ×400).

## The Study

From January 2011 through October 2011, a total of 455 samples (fecal, intestine, and milk) were collected from 57 farms in 12 provinces of China. All samples were evaluated by reverse transcription PCR (RT-PCR), by using previously described primers (*6*). Forty-five (78.95%) of the farms had at least 1 PEDV-positive sample. A total of 278 (61.11%) samples were PEDV positive, including 253 (of 402; 62.94%) fecal samples, 20 (of 31; 64.52%) intestine samples, and 5 (of 22; 22.73%) milk samples. The representative detection of PEDV in fecal samples of PED-affected farms is shown in Technical Appendix Figure 1.

Nine diarrhea samples were collected from pigs at 9 farms (where animals had severe diarrhea and mortality rate was high) for sequencing analysis of the full-length S gene (Technical Appendix Table 1). RT-PCR gene-specific primers were designed on the basis of the sequence of PEDV-CV777 strain (GenBank accession no. AF353511.1) (Table 1) and used to amplify 3 overlapping cDNA fragments spanning the entire S gene. The amplicons were sequenced in both directions (GenScript Co., Nanjing, PRC).

**Table 1:** Primers used in study of PEDV, China, 2011*

Primer name	Nucleotide sequence, 5′ → 3′	Primer location†
PEDVS1F	GGTAAGTTGCTAGTGCGTAA	20,570–20,589
PEDVS1R	CAGGGTCATCACAATAAAGAA	22,010–22,030
PEDVS2F	TTTCTGGACCGTAGCATC	21,939–21,956
PEDVS2R	TCCTGAAGTGGGACATAG	22,917–22,935
PEDVS3F	GAGTTGCCTGGTTTCTTC	22,816–22,833
PEDVS3R	TATAATTGCGCCTCAAAG	24,979–24,996

*PEDV, porcine epidemic diarrhea virus; F, forward; R, reverse. †Numbers correspond to the nucleotide positions within the CV777 genome.

The 9 PEDV S gene sequences were aligned with the sequences of 24 previously published PEDV S genes (Table 2) by using the ClustalX (version 1.82), Bioedit (version 7.0.9.0) and MegAlign version 5.0 (DNAStar Inc., Madison, WI, USA) software packages (*14*). The full-length S gene sequences of the 9 isolates from our study showed overall high conservation with the reference strains, up to 94.9%–99.6% homology (Technical Appendix Table 2). By phylogenetic analysis, 4 of the field isolates (CH2, CH5, CH6, CH7) clustered with the previously described strain JS-2004–2 from China. Three field isolates (CH1, CH8, CHGD-01) formed a unique cluster with the sequence-confirmed variant strain CH-FJND-3, which had been isolated from China in 2011 (*7*). CH1 and CH8 were isolated from 2 farms, where all sucking piglets had died from diarrhea, even though all of the sows had been vaccinated with the PEDV-CV777 strain–based inactivated vaccine. The isolated variant strains, CHGD-01 and CH1, were tested in experimental infection studies and found to cause illness in 100% of sucking piglets (data not shown).

**Table 2:** Isolates and reference strains used in study of porcine epidemic diarrhea virus outbreak, China, 2011

Virus strain	Country and year of isolation	GenBank accession no.	Reference
CH1	China 2011	JQ239429	This study
CH2	China 2011	JQ239430	This study
CH3	China 2011	JQ239431	This study
CH4	China 2011	JQ239432	This study
CH5	China 2011	JQ239433	This study
CH6	China 2011	JQ239434	This study
CH7	China 2011	JQ239435	This study
CH8	China 2011	JQ239436	This study
CHGD-01	China 2011	JN980698	This study
CH-FJND-1	China 2011	JN543367.1	Unpublished
CH-FJND-2	China 2011	JN315706.1	Unpublished
CH-FJND-3	China 2011	JN381492.1	(*7*)
JS-2004–2	China 2004	AY653204	Unpublished
LJB/03	China 2006	DQ985739	Unpublished
LZC	China 2006	EF185992	Unpublished
DX	China 2007	EU031893	Unpublished
CHS	China 1986	JN547228.1	(*8*)
Chinju99	South Korea 1999	AY167585	(*9*)
Spk1	South Korea 2002	AF400215	(*10*)
Parent DR13	South Korea 2006	DQ862099	(*11*)
Attenuated DR13	South Korea, 2006	DQ462404.2	(*11*)
KNU-0801	South Korea, 2008	GU180142	(*2*)
KNU-0802	South Korea, 2008	GU180143	(*2*)
KNU-0901	South Korea, 2009	GU180144	(*2*)
KNU-0902	South Korea, 2009	GU180145	(*2*)
KNU-0903	South Korea, 2009	GU180146	(*2*)
KNU-0904	South Korea, 2009	GU180147	(*2*)
KNU-0905	South Korea, 2009	GU180148	(*2*)
Br1/87	Great Britain, 1993	Z25483	(*12*)
MK	Japan, 1996	AB548624.1	(*3*)
NK	Japan	AB548623.1	(*3*)
Kawahira	Japan	AB548622.1	(*3*)
CV777	Belgium, 1988	AF353511	(*13*)

**Figure 2:**
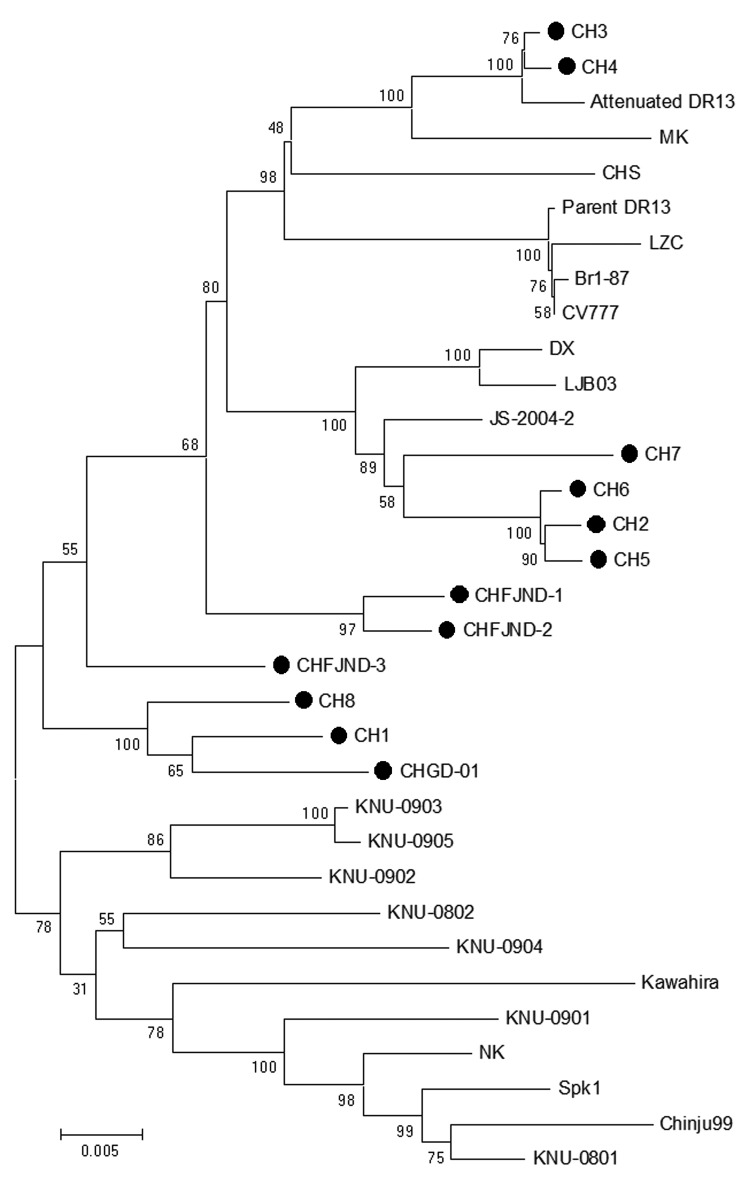
Phylogenetic trees of porcine epidemic diarrhea virus (PEDV) strains generated by the neighbor-joining method with nucleotide sequences of the full-length spike genes. Bootstrapping with 1,000 replicates was performed to determine the percentage reliability for each internal node. Horizontal branch lengths are proportional to genetic distances between PEDV strains. Black circles indicate PEDV field isolates from the 2011 outbreak in China. Scale bar indicates nucleotide substitutions per site.

The phylogenetic analysis of the S gene nucleotide sequences revealed 3 major clusters (Figure 2). Clade 1 comprised 6 strains from our study (CH2, CH3, CH4, CH5, CH6, CH7), the vaccine strain CV777 from China, the attenuated strain DR13 from South Korea, and 2 strains (CHFJND-1, CHFJND-2) that had been isolated in China in 2011. Clade 2 consisted of 4 variant strains (CH1, CH8, CHFJND-3, CHGD-01) that were identified from China in 2011. Clade 3 was composed of 9 isolates from South Korea and 2 strains from Japan (NK and Kawahira). The deduced amino acids of the 4 variant strains in clade 2 had 93% homology to CV777. Furthermore, the 4 variant strains from China (CH1, CH8, CHGD-01, CH-FJND-3) and 9 PEDV isolates from South Korea shared a 5-aa insertion (at positions 56–60 of the S protein) with CV777. One amino acid insertion at position 141 was shared among all variant strains and 6 isolates from South Korea (Technical Appendix Figure 2). In the S genes, 132 point mutations were found that accounted for genetic diversity among the isolates.

The recent 4 isolates from China (CH2, CH5, CH6, CH7) were closely related to the previously identified isolates from China (JS-2004–2, LJB03, DX) and another 4 variant strains. Three of the new isolates (CH1, CH8, CHGD-01) were highly pathogenic in piglets. All strains were obtained from farms that used the CV777-based inactivated vaccine but had 100% prevalence of diarrhea in pigs (Technical Appendix Table 1). Another 2 field isolates (CH3, CH4) from 2 farms with pigs with severe diarrhea shared the highest sequence identity with attenuated strain DR13 from South Korea (99.2% and 99.1%, respectively), which has been in routine use as an oral vaccine against PEDV in South Korea since 2004 (*15*). The appearance of strains in China similar to those from South Korea and their role in the recent PEDV outbreak should be further investigated.

## Conclusions

RT-PCR amplification and sequencing analysis of the full-length PEDV spike genes were used to investigate isolates from diarrhea samples from local pig farms with severe diarrhea in piglets. Both classical and variant strains were detected, implying a diverse distribution profile for PEDV on pig farms in China. The sequence insertions and mutations found in the variant strains may have imparted a stronger pathogenicity to the new PEDV variants that influenced the effectiveness of the CV777-based vaccine, ultimately causing the 2011 outbreak of severe diarrhea on China’s pig farms. Future studies should investigate the biologic role of these particular insertions and mutations. Furthermore, our study of the full-length S gene revealed a more comprehensive distribution profile that reflects the current PEDV status in pig farms in China, including the presence of a strain similar to strain DR13, isolated in South Korea. Collectively, these data indicate the urgent need to develop novel variant strain–based vaccines to treat the current outbreak in China.

Technical Appendix Results from samples from pig farms where porcine epidemic diarrhea virus (PEDV) was found, China, 2011; nucleotide sequence similarity based on the full-length spike genes of China PEDV field isolates and PEDV reference strains; reverse transcription PCR with primers specific for spike genes from PEDV strains; and alignment of deduced amino acid sequences of the spike proteins of PEDV field isolates and reference strains.
