# Supplementary material for: New Variants of Porcine Epidemic Diarrhea Virus, China, 2011
Source: Emerg Infect Dis. 2012 Aug;18(8):1350–3. doi: 10.3201/eid1808.120002 (PMC3414035; doi:10.3201/eid1808.120002)
Supplement: Technical Appendix — Results from samples from pig farms where porcine epidemic diarrhea virus (PEDV) was found, China, 2011; nucleotide sequence similarity based on the full-length spike genes of China PEDV field isolates and PEDV reference strains; reverse transcription PCR with primers specific for spike genes from PEDV strains; and alignment of deduced amino acid sequences of the spike proteins of PEDV field isolates and reference strains. [file 12-0002_Techapp-s1.pdf]

Article DOI: http://dx.doi.org/10.3201/eid1803.120002

New Variants of Porcine Epidemic Diarrhea Virus, China, 2011

Technical Appendix

Technical Appendix Table 1. Results from samples from pig farms where PEDV was found, China, 2011

Strain	District	Collection date	No. sows	Vaccination†	Illness rate,%/y‡
CH1	Hubei	2011 Jan	800	Yes	60
CH2	Henan	2011 Feb	1,000	Yes	80
CH3	Anhui	2011 Apr	200	Yes	90
CH4	Jiangxi	2011 Mar	100	Yes	100
CH5	Sichuan	2011 Jun	600	Yes	70
CH6	Zhejiang	2011 Jul	1,000	Yes	90
CH7	Shandong	2011 Mar	900	Yes	85
CH8	Guangxi	2011 Sep	1,200	Yes	60
CHGD-01	Guangdong	2011 Feb	8,000	Yes	80

*PEDV, porcine epidemic diarrhea virus.

†Sows were vaccinated with divalent inactivated transmissible gastroenteritis and PEDV vaccine before delivery. All samples were collected during the 2011 epidemic.

‡The mortality rate for all infected animals was 100%.

Technical Appendix Table 2. Nucleotide sequence similarity based on the full-length spike genes of China PEDV field isolates and PEDV reference strains*

	CH4	1	2	CH1	CH2	CH5	CH6	CH7	CH8	3	4	5	6	7	8	9	10	11	12	13	14	15	16	17	18	19	20	21	22	23	24	25	CH3
CH4	***	99.4	96.7	93.9	95.9	95.8	96	95.8	94.1	97.5	96.7	94.9	93.7	93.8	96.6	96.8	95.9	96.3	94.6	94.5	93.9	94.4	93.8	93.9	93.4	93.8	95.8	96.3	97.6	95	96.9	94.4	99.7
1	0.6	***	96.5	93.7	95.7	95.6	95.8	95.6	94	97.3	96.5	94.7	93.4	93.6	96.3	96.6	95.8	96.1	94.4	94.3	93.8	94.1	93.6	93.7	93.2	93.6	95.6	96	97.3	94.8	96.6	94.2	99.5
2	3.3	3.6	***	93.9	95.6	95.6	95.8	95.3	94.1	96.3	95.9	94.5	94	94.3	96.2	99.9	95.9	96.2	94.1	94.3	94.4	94.1	94	94.1	93.9	94	95.8	99.3	95.9	94.9	99.8	94.2	96.7
CH1	6.4	6.6	6.4	***	95.1	95.1	95.1	96.4	98.5	94.7	95.6	97.1	98.1	93.6	93.6	93.9	95.2	95.5	94.2	94.5	96.2	94.8	96.6	95.5	94.9	95.4	95.1	93.4	93.6	94.7	94	94.3	94
CH2	4.3	4.5	4.5	5.1	***	99.5	99.6	97.7	95.8	96.7	96.1	94.9	94.5	92.6	95.5	95.7	97.3	98.4	93.2	93.2	94.1	93.5	94.4	94.3	93.4	94.2	97.2	95.2	95.2	93.8	95.8	93.1	95.9
CH5	4.3	4.5	4.6	5.1	0.5	***	99.6	97.6	95.8	96.7	96.1	94.8	94.5	92.6	95.6	95.7	97.3	98.3	93.1	93.1	94	93.5	94.4	94.3	93.3	94.3	97.2	95.2	95.1	93.8	95.7	93.1	95.9
CH6	4.2	4.4	4.4	5	0.4	0.4	***	97.7	95.9	96.9	96.3	95	94.6	92.8	95.7	95.9	97.4	98.5	93.3	93.3	94.2	93.7	94.5	94.4	93.5	94.4	97.3	95.4	95.2	94	95.9	93.3	96
CH7	4.4	4.6	4.8	3.7	2.4	2.4	2.3	***	95.2	96.5	95.9	94.6	95.3	92.4	95.3	95.4	97.2	97.8	92.9	93	93.9	93.2	94.1	94.1	93.4	94	97.1	94.9	95.1	93.5	95.5	92.9	95.9
CH8	6.1	6.3	6.2	1.6	4.3	4.3	4.3	4.9	***	95.1	95.9	97.4	97.4	93.9	93.9	94.2	95.4	96.2	94.5	94.8	96.4	95.1	96.9	95.8	95	95.7	95.3	93.7	93.8	95	94.2	94.5	94.2
3	2.6	2.8	3.8	5.5	3.4	3.4	3.2	3.6	5.1	***	99	97.2	94.8	93	96	96.4	96.8	97.3	93.6	93.6	94.5	93.5	94.6	94.6	93.7	94.5	96.7	95.9	96	94.2	96.4	93.5	97.6
4	3.4	3.6	4.3	4.5	4	4	3.8	4.2	4.2	1	***	98.2	95.7	93.7	95.4	96	96.3	96.7	94.1	94.2	95.3	94.1	95.6	95.2	94.2	95.1	96.2	95.4	95.5	94.7	95.9	94.1	96.8
5	5.4	5.5	5.7	3	5.3	5.4	5.2	5.6	2.6	2.9	1.9	***	97.1	94.2	94.2	94.6	95.1	95.5	94.8	95.1	96.5	95.1	96.9	95.9	95	95.8	95	94	94.1	95.2	94.5	94.7	94.9
6	6.6	6.9	6.3	1.9	5.7	5.7	5.6	4.8	2.7	5.4	4.4	3	***	93.8	93.4	94	95	95.3	94	94.4	96.2	94.6	96.4	95.3	94.5	95.2	94.9	93.5	93.3	94.4	94	94.1	93.8
7	6.5	6.7	5.9	6.7	7.8	7.9	7.7	8.1	6.4	7.3	6.7	6	6.6	***	93.5	94.3	93	93.1	94.2	98	94.5	96.3	94.1	94.4	94.2	94.3	92.9	93.8	93.3	97	94.3	97.3	93.9
8	3.5	3.8	3.9	6.8	4.6	4.6	4.5	4.9	6.4	4.2	4.7	6.1	6.9	6.9	***	96.3	95.9	96.2	93.8	94	94.1	94.3	94.3	94.3	93.8	94.2	95.8	95.8	95.7	94.7	96.3	94	96.7
9	3.2	3.5	0.1	6.4	4.4	4.5	4.3	4.7	6.1	3.7	4.2	5.7	6.2	5.9	3.8	***	96	96.3	94.1	94.4	94.4	94.2	94	94.2	93.9	94.1	95.9	99.4	96	94.9	99.9	94.3	96.8
10	4.2	4.4	4.3	5	2.8	2.8	2.7	2.9	4.8	3.3	3.8	5.1	5.2	7.4	4.2	4.2	***	98.1	93.5	93.5	94.4	93.6	94.7	94.8	93.9	94.7	99.1	95.4	95.3	94	96	93.4	96
11	3.8	4	3.9	4.7	1.7	1.7	1.6	2.2	3.9	2.8	3.4	4.7	4.9	7.2	3.9	3.8	1.9	***	93.6	93.7	94.8	94	95.2	95.1	94.1	95.1	98	95.8	95.6	94.3	96.3	93.6	96.3
12	5.6	5.9	6.2	6	7.2	7.3	7.1	7.5	5.7	6.7	6.2	5.4	6.3	6	6.5	6.1	6.9	6.7	***	95.1	94.6	94.8	94.3	94.6	94.2	94.5	93.4	93.7	94	95.5	94.2	95	94.6
13	5.7	6	5.9	5.7	7.2	7.3	7	7.4	5.5	6.7	6.1	5.1	5.8	2	6.3	5.9	6.9	6.7	5.1	***	95.4	97.9	94.8	95.1	95.1	95.1	93.4	93.8	93.9	98.2	94.4	98.6	94.6
14	6.4	6.6	5.9	3.9	6.2	6.2	6.1	6.4	3.7	5.8	4.9	3.6	3.9	5.7	6.2	5.8	5.9	5.4	5.6	4.8	***	95.7	96.5	95.8	96.2	95.7	94.3	93.9	93.3	95.4	94.4	95	94
15	5.9	6.1	6.1	5.4	6.9	6.9	6.7	7.1	5	6.8	6.1	5.1	5.6	3.8	5.9	6.1	6.7	6.3	5.5	2.1	4.4	***	95.3	95.6	95.8	95.5	93.6	93.6	93.7	97	94.2	97.1	94.4
16	6.5	6.7	6.3	3.5	5.9	5.9	5.8	6.2	3.2	5.6	4.6	3.2	3.7	6.1	6	6.2	5.5	5	5.9	5.4	3.6	4.8	***	97.8	95.4	97.6	94.7	93.5	93.3	94.9	94	94.6	93.9
17	6.4	6.6	6.2	4.7	6	6	5.9	6.2	4.3	5.6	5	4.3	4.9	5.8	5.9	6.1	5.5	5.1	5.6	5	4.4	4.6	2.2	***	96.8	99.8	94.6	93.6	93.3	95.4	94.1	95.2	94
18	6.9	7.2	6.4	5.3	7	7	6.8	6.9	5.2	6.6	6	5.2	5.7	6	6.5	6.3	6.4	6.1	6.1	5.1	3.9	4.4	4.8	3.3	***	96.7	93.8	93.4	92.7	95.4	93.9	95.1	93.5
19	6.5	6.7	6.2	4.8	6	6	5.9	6.3	4.4	5.7	5.1	4.4	4.9	5.9	6	6.2	5.5	5.1	5.8	5.1	4.5	4.7	2.4	0.2	3.4	***	94.6	93.6	93.2	95.3	94	95.1	93.9
20	4.3	4.5	4.3	5.1	2.9	2.9	2.8	3	4.8	3.4	3.9	5.2	5.3	7.5	4.3	4.2	0.9	2	7	6.9	5.9	6.7	5.5	5.6	6.5	5.7	***	95.4	95.1	93.9	95.9	93.3	95.9
21	3.8	4.1	0.7	7	5	5	4.8	5.3	6.7	4.2	4.7	6.3	6.8	6.5	4.3	0.6	4.7	4.3	6.6	6.5	6.4	6.7	6.8	6.7	6.9	6.8	4.8	***	95.5	94.4	99.3	93.7	96.3
22	2.5	2.7	4.2	6.8	5	5.1	5	5.1	6.5	4.1	4.7	6.2	7.1	7	4.4	4.1	4.9	4.5	6.3	6.4	7	6.7	7	7	7.7	7.1	5.1	4.7	***	94.5	96	93.9	97.6
23	5.2	5.4	5.3	5.5	6.5	6.5	6.3	6.8	5.3	6.1	5.5	5	5.8	3.1	5.5	5.3	6.3	6	4.7	1.8	4.8	3	5.3	4.8	4.8	4.9	6.4	5.9	5.7	***	94.9	98.2	95.1
24	3.2	3.5	0.2	6.3	4.4	4.4	4.2	4.7	6.1	3.7	4.2	5.7	6.2	6	3.8	0.1	4.2	3.8	6.1	5.9	5.9	6.1	6.3	6.2	6.4	6.2	4.2	0.7	4.1	5.3	***	94.3	96.9
25	5.8	6.1	6.1	6	7.3	7.3	7.1	7.5	5.7	6.9	6.2	5.5	6.1	2.8	6.3	6	6.9	6.7	5.2	1.4	5.2	2.9	5.6	5	5.1	5.1	7	6.6	6.4	1.8	6	***	94.5
CH3	0.3	0.5	3.3	6.3	4.3	4.3	4.1	4.3	6.1	2.5	3.3	5.3	6.5	6.4	3.4	3.2	4.1	3.8	5.6	5.6	6.2	5.8	6.4	6.3	6.8	6.4	4.2	3.8	2.4	5.1	3.2	5.7	***

*PEDV, porcine epidemic diarrhea virus; 1, attenuated DR13; 2, Br1-87; 3, CHFJND-1; 4, CH-FJND-2; 5, CH-FJND-3; 6, CHGD-01; 7, Chinju99; 8, CHS; 9, CV777; 10, DX; 11, JS-2004-2; 12, Kawahira; 13, KNU-0801; 14, KNU-0802; 15, KNU-0901; 16, KNU-0902; 17, KNU-0903; 18, KNU-0904; 19, KNU-0905; 20, LJB03; 21, LZC; 22, MK; 23, NK; 24, parent DR13; 25, Spk1.

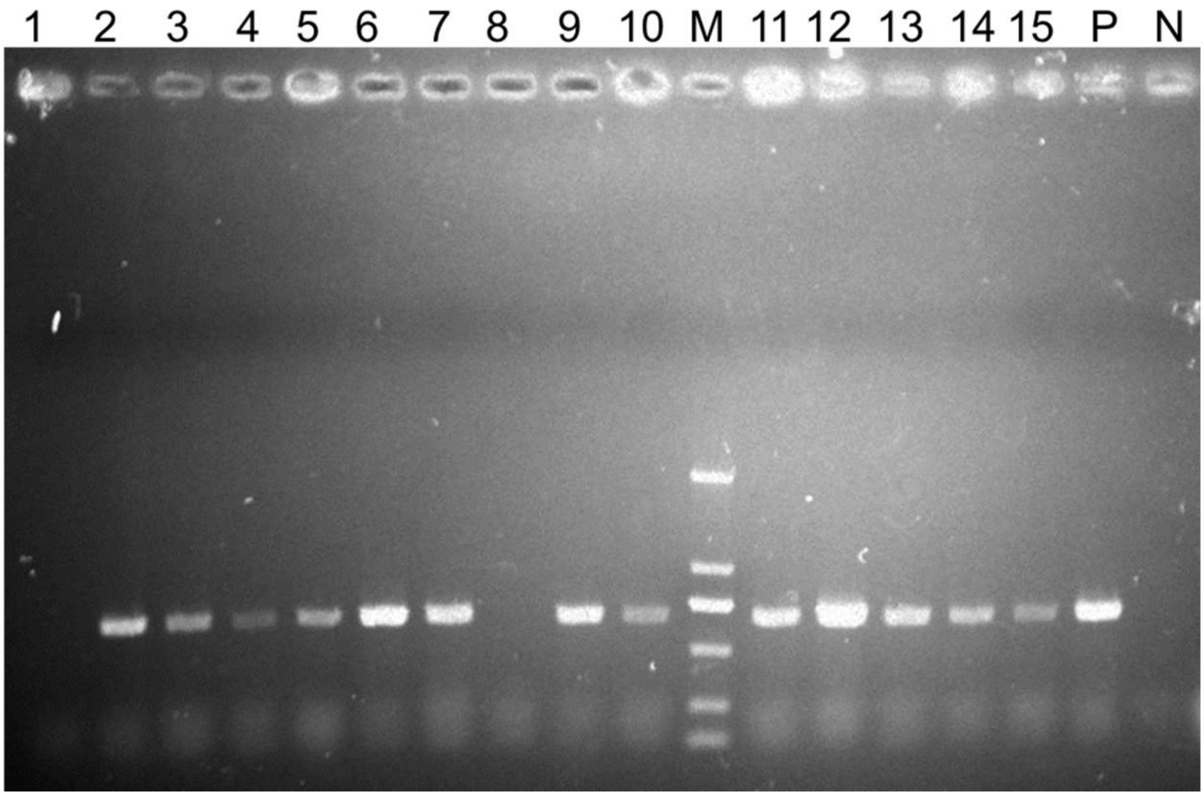

Technical Appendix Figure 1. A total of 15 fecal samples were collected from suckling piglets of a single farrowing house in Hubei province and analyzed by reverse transcription PCR with primers specific for spike genes from porcine epidemic diarrhea virus (PEDV) strains. The expected product size was 667 bp. PEDV was detected in 13 (86.7%) of 15 samples. Lanes 1–15, clinical fecal samples; M, marker 2000; P, positive control; N, negative control.

Technical Appendix Figure 2 (below and following pages). Alignment of deduced amino acid sequences of the spike proteins of porcine epidemic diarrhea virus field isolates and porcine epidemic virus reference strains. Dots indicate amino acids that are identical to those in CV777. Dashes indicate deleted sequences. A-DR13, attenuated DR13; P-DR13, parent DR13.

Strain	2	3	5	10	15	27	28	29	30	56	57	58	59	60	61	65	69	70	71	72	73	75	85	87
CV777	R	S	I	L	P	Q	S	T	T	-	-	-	-	-	M	S	G	T	G	I	E	A	Y	D
CH1	K	.	T	F	S	S	A	N	.	G	E	N	Q	G	V	T	A	G	Q	H	P	.	H	R
CH8	K	.	T	F	S	S	A	N	.	G	E	N	Q	G	V	T	A	G	Q	H	P	.	H	R
CHGD-01	.	.	.	F	S	S	A	N	.	G	E	N	Q	G	V	T	A	G	Q	H	P	.	H	R
CH-FJND-3	K	.	T	F	S	S	A	N	.	G	E	N	Q	G	V	T	A	G	Q	H	P	.	H	R
KNU-0802	.	.	.	F	.	S	A	N	.	G	E	N	Q	G	V	T	A	G	Q	H	P	.	H	R
CH7	K	.	T	.	S	.	.	.	I	-	-	-	-	-	.	.	.	.	.	L	.	.	.	.
CH6	K	.	T	.	.	.	.	.	I	-	-	-	-	-	.	.	.	.	.	L	.	.	.	.
CH2	K	.	T	.	S	.	.	.	I	-	-	-	-	-	.	.	.	.	.	L	.	.	.	.
CH5	K	.	T	.	S	.	.	.	I	-	-	-	-	-	.	.	.	.	.	L	.	.	.	.
CH4	T	P	.	F	L	.	.	.	I	-	-	-	-	-	.	.	.	.	.	.	.	D	.	.
CH3	T	P	.	F	L	.	.	.	I	-	-	-	-	-	.	.	.	.	.	.	.	D	.	.
JS-2004-2	K	.	T	.	S	.	.	.	I	-	-	-	-	-	.	.	.	.	.	L	.	.	.	.
Br1-87	.	.	.	.	.	.	.	.	.	-	-	-	-	-	.	.	.	.	.	.	.	.	.	.
CH-FJND-1	T	P	.	F	L	.	.	.	I	-	-	-	-	-	.	.	.	.	.	.	.	D	.	.
CH-FJND-2	T	P	.	F	L	.	.	.	I	-	-	-	-	-	.	.	.	.	.	.	.	D	.	.
CHS	K	.	T	F	S	.	.	.	.	-	-	-	-	-	.	.	.	.	.	.	.	.	.	.
MK	K	.	.	F	S	.	T	.	.	-	-	-	-	-	.	.	.	.	.	.	.	D	.	.
NK	K	.	.	F	.	R	A	K	.	G	E	Q	Q	D	G	R	.	G	Q	H	.	.	H	K
Kawahira	K	F	.	F	.	S	A	K	.	G	E	Q	Q	N	G	P	A	G	T	H	T	.	H	K
Chinju99	.	.	.	F	.	S	A	N	.	-	V	K	H	A	G	-	A	A	D	.	Q	.	H	R
KNU-0903	.	.	T	F	.	S	A	V	.	G	E	K	Q	V	D	R	A	G	R	Y	D	.	H	R
KNU-0901	.	.	T	F	.	S	A	N	.	G	E	T	Q	A	G	P	A	G	R	H	P	.	H	R
Spk1	.	.	.	S	.	S	A	R	.	G	E	T	Q	G	G	R	A	G	R	H	.	.	H	R
DX	K	.	T	F	S	.	.	.	I	-	-	-	-	-	.	.	.	.	.	L	.	.	.	.
LZC	.	.	.	.	.	.	.	.	.	-	-	-	-	-	.	.	.	.	.	.	.	.	.	.
LJB03	K	.	T	F	S	.	.	.	I	-	-	-	-	-	.	.	.	.	.	L	.	.	.	.
P-DR13	.	.	.	.	.	.	.	.	.	-	-	-	-	-	.	.	.	.	.	.	.	.	.	.
A-DR13	T	P	.	F	L	.	.	.	I	-	-	-	-	-	.	.	.	.	.	.	.	D	.	.

Strain	88	90	119	121	131	132	139	141	151	153	158	159	160	163	164	165	180	185	188	196	198	202
CV777	S	Q	N	I	D	N	V	-	F	K	Y	M	R	K	D	I	A	H	L	A	R	R
CH1	G	H	.	T	S	I	A	N	.	.	H	.	S	E	H	S	S	Y	F	.	K	S
CH8	G	H	.	T	S	I	A	N	S	.	H	.	S	E	H	S	P	Y	F	.	K	S
CHGD-01	G	H	.	T	S	I	A	N	.	.	H	.	S	E	H	S	S	Y	F	.	K	S
CH-FJND-3	G	H	.	T	S	I	A	N	.	.	H	.	S	E	H	S	S	.	F	.	K	S
KNU-0802	G	H	.	T	S	S	A	D	.	.	H	.	S	E	H	S	S	.	F	.	K	S
CH7	.	.	.	.	.	.	.	-	.	R	.	.	Q	.	N	.	.	.	.	.	.	K
CH6	.	.	.	.	.	.	.	-	.	R	.	.	Q	.	N	.	.	.	.	V	.	K
CH2	.	.	.	.	.	.	.	-	.	R	.	.	Q	.	N	.	.	.	.	V	.	K
CH5	.	.	.	.	.	.	.	-	.	R	.	.	Q	.	N	.	.	.	.	V	.	K
CH4	.	.	S	.	.	.	.	-	.	.	-	L	Q	.	N	.	.	.	I	.	.	K
CH3	.	.	S	.	.	.	.	-	.	.	-	L	Q	.	N	.	.	.	I	.	.	K
JS-2004-2	A	.	.	.	.	.	.	-	.	R	.	.	Q	.	N	.	.	.	.	.	.	K
Br1-87	.	.	.	T	.	.	.	-	.	.	.	.	.	.	.	.	.	.	.	.	.	.
CH-FJND-1	.	.	S	.	.	.	.	-	S	.	-	L	Q	.	N	.	.	.	I	.	.	K
CH-FJND-2	.	.	S	.	.	.	.	-	.	.	-	H	M	E	H	S	S	.	F	.	K	S
CHS	.	.	.	.	.	.	.	-	.	.	.	L	Q	.	N	.	.	.	.	.	.	K
MK	.	.	.	.	.	.	A	-	.	.	H	.	Q	R	H	.	.	.	I	.	.	K
NK	G	H	.	T	S	.	A	D	.	.	H	.	S	E	H	S	.	.	.	.	K	S
Kawahira	G	H	.	T	S	.	A	D	.	.	H	.	S	E	H	S	.	.	.	.	K	S
Chinju99	G	H	.	T	S	S	A	-	.	.	H	.	S	.	H	S	.	.	.	.	N	S
KNU-0903	G	H	.	T	S	.	A	D	.	.	H	.	S	E	H	S	.	.	.	.	K	S
KNU-0901	G	H	.	T	S	S	A	D	.	.	H	.	S	E	H	S	.	.	.	.	N	S
Spk1	G	H	.	T	S	.	A	D	.	.	H	.	S	E	H	S	.	.	.	.	K	S
DX	.	R	.	.	N	.	.	-	.	.	H	.	Q	.	N	.	.	.	.	.	.	K
LZC	.	.	.	.	.	.	.	-	.	F	.	.	.	.	.	.	.	.	.	.	.	.
LJB03	.	.	.	.	N	.	.	-	.	.	H	.	Q	.	N	.	.	.	.	.	.	K
P-DR13	.	.	.	.	.	.	.	-	.	.	.	.	.	.	.	.	.	.	.	.	.	.
A-DR13	.	.	S	.	.	.	.	-	.	.	-	L	Q	.	N	T	.	.	I	.	.	K

Strain	203	204	212	217	229	231	238	248	249	272	288	290	304	315	316	330	344	357	360	362	364	369	371
CV777	R	S	T	M	Y	E	T	D	S	L	L	I	H	V	D	S	L	D	L	I	A	A	E
CH1	G	G	E	T	S	Q	I	E	P	V	.	.	Q	L	Q	.	.	.	.	T	.	.	Q
CH8	G	G	E	T	S	Q	I	E	P	V	.	.	Q	L	Q	.	.	.	.	T	.	.	Q
CHGD-01	G	G	E	.	S	Q	I	E	P	V	.	.	Q	.	Q	.	.	.	.	T	.	.	Q
CH-FJND-3	G	G	E	.	S	Q	I	E	P	V	.	M	Q	.	Q	.	F	N	.	T	.	.	Q
KNU-0802	G	G	E	.	S	Q	I	E	.	V	.	.	Q	A	Q	.	.	N	.	T	.	.	Q
CH7	.	.	.	.	.	.	S	.	.	.	.	.	Q	A	Q	.	.	E	.	T	.	.	Q
CH6	.	.	.	.	.	.	S	.	.	.	.	.	Q	.	R	.	.	N	.	T	.	.	Q
CH2	.	.	.	.	.	.	S	.	.	.	.	.	Q	.	Q	.	.	N	.	T	.	.	Q
CH5	.	.	.	.	.	.	S	.	.	.	.	.	Q	.	Q	.	.	N	.	T	.	.	Q
CH4	.	.	.	.	.	.	S	.	.	.	R	.	Q	A	Q	F	.	.	K	.	T	V	.
CH3	.	.	.	.	.	.	S	.	.	.	W	.	Q	A	Q	F	.	.	K	.	T	V	.
JS-2004-2	.	.	.	.	.	.	S	.	.	.	.	.	Q	A	Q	.	.	.	.	T	.	.	Q
Br1-87	.	.	.	.	.	.	.	.	.	.	.	.	.	.	.	.	.	.	.	.	.	.	.
CH-FJND-1	.	.	.	.	.	.	S	.	.	V	.	M	Q	.	Q	.	F	N	.	T	.	.	Q
CH-FJND-2	G	G	E	.	S	Q	I	E	P	V	.	M	Q	.	Q	.	F	N	.	T	.	.	Q
CHS	K	.	.	.	.	.	S	.	A	.	.	.	.	.	N	.	.	.	S	.	.	.	Q
MK	.	.	.	.	.	.	S	E	.	.	W	.	Q	A	Q	.	.	N	K	.	T	V	.
NK	G	G	E	.	S	Q	I	.	.	F	W	.	Q	A	Y	.	.	.	S	.	S	.	Q
Kawahira	G	G	E	.	S	Q	I	E	.	.	W	.	Q	.	Q	.	.	N	S	.	.	.	.
Chinju99	G	G	E	.	S	Q	I	E	.	F	W	.	Q	A	Y	.	.	.	K	.	S	.	Q
KNU-0903	G	G	K	.	S	Q	I	E	P	.	W	.	Q	A	Q	.	.	.	.	T	.	.	Q
KNU-0901	G	G	E	.	S	Q	I	E	.	F	W	.	Q	A	Y	.	.	.	K	.	S	.	Q
Spk1	G	G	E	.	S	Q	I	E	.	F	W	.	Q	A	Y	.	.	.	K	.	S	.	Q
DX	.	.	.	.	.	.	S	.	.	.	.	.	Q	A	Q	.	.	.	.	T	.	.	Q
LZC	.	.	.	.	.	.	.	.	.	.	.	.	.	.	.	.	.	.	.	.	.	.	.
LJB03	.	.	.	.	.	.	S	.	.	.	.	.	Q	A	Q	.	.	.	.	T	.	.	Q
P-DR13	.	.	.	.	.	.	.	.	.	.	.	.	.	.	.	.	.	.	.	.	.	.	.
A-DR13	.	.	.	.	.	.	S	.	.	.	W	.	Q	A	Q	F	.	.	K	.	T	V	.

Strain	384	399	444	460	480	502	523	527	529	533	555	569	573	600	611	614	619	622	643	645	665	677	717
CV777	N	R	I	S	A	I	A	L	S	V	T	K	S	G	A	S	G	L	E	I	K	I	N
CH1	.	.	V	A	.	T	S	H	G	I	S	.	.	S	E	.	.	F	.	V	.	F	D
CH8	.	.	V	A	.	T	S	H	G	I	S	.	.	S	E	.	.	F	.	V	.	F	D
CHGD-01	.	.	.	A	S	T	.	H	G	I	S	.	.	S	E	.	.	F	.	V	.	F	D
CH-FJND-3	.	.	.	A	.	.	S	P	G	I	S	.	G	S	E	G	.	F	.	V	R	F	D
KNU-0902	.	.	.	A	.	T	S	H	G	.	R	N	.	S	D	.	.	F	.	V	.	F	D
CH7	.	.	V	A	.	.	S	H	G	I	L	.	.	S	E	.	A	F	.	V	.	F	D
CH6	.	.	V	A	.	.	S	H	G	I	S	N	T	S	.	.	V	F	.	V	.	F	D
CH2	.	.	V	A	.	.	S	H	G	I	S	N	T	S	.	.	V	F	.	V	.	F	D
CH5	.	.	V	A	.	.	S	H	G	I	S	N	T	S	.	.	V	F	.	V	.	F	D
CH4	K	K	V	A	S	.	.	H	G	I	.	.	.	.	E	.	.	F	Q	V	.	F	D
CH3	K	K	V	A	S	.	.	H	G	I	.	.	.	.	E	.	.	F	Q	V	.	F	D
JS-2004-2	.	.	.	A	.	.	S	H	G	I	S	.	.	S	E	.	.	F	.	V	.	F	D
Br1-87	.	.	.	.	.	.	.	.	.	.	.	.	.	.	.	.	.	.	.	.	.	.	.
CH-FJND-1	.	.	.	A	.	.	S	P	G	I	S	.	.	S	E	G	.	F	.	V	R	F	D
CH-FJND-2	.	.	.	A	.	.	S	P	G	I	S	.	.	S	E	G	.	F	.	V	R	F	D
CHS	.	K	V	A	S	V	.	S	G	.	.	N	.	.	E	.	S	F	.	V	.	F	D
MK	.	K	V	A	S	.	.	H	G	I	.	.	.	.	E	.	.	.	.	V	.	F	D
NK	.	.	.	A	S	.	.	S	G	.	.	.	.	.	.	.	.	F	.	V	.	F	D
Kawahira	K	K	V	A	S	.	.	H	G	I	R	N	.	.	E	.	V	F	.	V	.	F	.
Chinju99	.	.	.	A	S	.	.	S	G	.	R	.	.	.	.	.	.	Y	N	L	.	.	.
KNU-0903	.	.	.	A	.	.	S	H	G	I	R	N	.	S	D	G	A	F	.	V	.	F	D
KNU-0901	.	.	.	A	S	.	.	S	G	.	R	.	.	.	.	.	.	F	Q	V	.	F	D
Spk1	.	.	.	A	S	.	.	S	G	.	R	.	.	.	.	.	.	F	.	V	.	F	D
DX	.	.	.	A	.	.	S	H	G	I	S	.	.	S	E	G	.	F	.	V	.	F	D
LZC	.	.	.	.	.	.	.	.	.	.	.	.	.	.	.	.	.	.	.	.	.	.	.
LJB03	.	.	.	A	.	.	S	H	G	I	S	.	.	S	E	.	.	F	.	V	.	F	D
P-DR13	.	.	.	.	.	.	.	.	.	.	.	.	.	.	.	.	.	.	.	.	.	.	.
A-DR13	K	K	V	A	.	.	.	H	G	I	.	.	.	.	.	E	.	.	.	Q	.	F	D

Strain	1172	1174	1177	1181	1183	1184	1203	1204	1210	1217	1225	1242	1247	1270	1275	1303	1308	1312	1340	1342	1369	1386
CV777	N	L	A	V	G	E	T	Y	F	F	S	S	D	T	P	T	R	N	I	V	G	A
CH1	.	I	.	.	D	.	-	D	S	Y	G	R	E	.	S	S	Q	Y	.	F	.	V
CH8	D	I	.	.	D	.	N	H	V	.	.	R	.	.	.	.	Q	Y	.	F	C	V
CHGD-01	.	I	.	.	D	.	-	N	.	Y	G	R	E	.	S	.	Q	Y	.	F	.	.
CH-FJND-3	.	I	D	I	.	D	.	.	L	.	.	R	.	.	.	.	.	Y	.	F	.	.
KNU-0902	.	I	.	.	.	D	.	H	.	.	.	.	.	.	.	I	.	Y	.	F	C	.
CH7	.	I	.	.	D	.	-	D	.	Y	G	R	E	.	S	S	Q	Y	.	F	.	V
CH6	D	I	.	.	D	.	N	H	.	.	.	R	.	.	.	.	Q	Y	V	F	C	V
CH2	D	I	.	.	D	.	N	H	.	.	.	R	.	.	.	.	Q	Y	V	F	C	V
CH5	D	T	.	.	D	.	N	H	.	.	.	R	.	.	.	.	Q	Y	V	F	C	V
CH4	.	I	D	.	.	D	.	.	.	.	.	.	.	I	.	.	.	Y	.	F	.	.
CH3	.	I	D	.	.	D	.	.	.	.	.	.	.	I	.	.	.	Y	.	F	.	.
JS-2004-2	D	I	.	.	D	.	N	H	.	.	.	R	.	.	.	.	Q	Y	.	F	C	.
Br1-87	.	.	.	.	.	.	.	.	.	.	.	.	.	.	.	.	.	.	.	.	.	.
CH-FJND-1	.	I	D	I	.	D	.	.	L	.	.	R	.	.	.	.	.	Y	.	F	.	.
CH-FJND-2	.	I	D	I	.	D	.	.	L	.	.	R	.	.	.	.	.	Y	.	F	.	.
CHS	.	T	.	.	.	D	.	H	.	.	.	.	.	.	.	.	.	Y	.	F	.	.
MK	.	I	.	.	.	.	.	.	.	.	.	.	.	.	S	.	.	Y	.	F	.	.
NK	.	I	.	.	.	D	.	H	.	.	.	.	.	.	.	.	.	Y	.	F	.	.
Kawahira	.	I	.	.	.	.	N	.	.	.	.	.	.	.	.	.	.	Y	.	F	.	.
Chinju99	.	I	.	.	.	D	.	.	.	.	.	.	.	.	.	.	.	Y	.	F	.	G
KNU-0903	.	T	.	.	D	D	.	H	.	.	.	.	.	.	.	.	.	Y	.	F	C	.
KNU-0901	.	I	.	.	.	D	.	H	.	.	.	.	.	.	.	.	.	Y	V	F	C	.
Spk1	.	I	.	.	.	D	.	H	.	.	.	.	.	.	.	.	.	Y	.	F	.	.
DX	D	I	.	.	D	.	N	.	.	.	.	R	.	.	.	.	Q	Y	.	F	.	.
LZC	.	.	.	.	.	.	.	.	.	.	.	.	.	.	.	.	.	.	.	.	.	.
LJB03	D	I	.	.	D	.	N	.	.	.	.	R	.	.	.	.	Q	Y	.	F	.	.
P-DR13	.	.	.	.	.	.	N	.	.	.	.	.	.	N	.	.	.	.	.	F	.	.
A-DR13	.	I	D	.	.	D	.	.	.	.	.	.	.	I	.	.	.	Y	.	F	.	.
